# *Clerodendranthus spicatus*: a comprehensive review of the chemical constituents, pharmacology, quality control and clinical applications

**DOI:** 10.3389/fphar.2025.1452797

**Published:** 2025-03-13

**Authors:** Xingmeng Jiao, Qiong Jin, Peifeng Zhu, Zhuomin Tan, Li Li, Lu Liu

**Affiliations:** ^1^ School of Traditional Chinese Medicine, Yunnan University of Chinese Medicine, Kunming, China; ^2^ College of Pharmacy, Kunming Medical University, Kunming, China

**Keywords:** *Clerodendranthus spicatus*, chemical constituents, pharmacology, review, quality control, clinical application

## Abstract

*Clerodendranthus spicatus* (CS), is an herbaceous perennial belonging to the family Lamiaceae. The herb is extensively employed in traditional Chinese medicine for the mitigation of nephritis, cystitis, kidney stones, urological tract stones, gout, and other urinary conditions. Numerous research studies have been conducted in the past to explore the traditional medicinal value, phytochemical composition, pharmacological effects, and quality control measures associated with this plant. This has motivated us to systematically search various online databases such as Google Scholar, PubMed, Science Direct, Elsevier, CNKI, Scopus, Embase, and Web of Science using specific keywords to get the most recent research information findings related to this plant. Phytochemical investigations have identified that this plant predominantly contains flavonoids, terpenoids, phenylpropanoids, and volatile oil compounds. Certain constituents have been employed as markers in quality assessment research, and some were recognized as bioactive agents in the management of specific ailments. These components have demonstrated notable effectiveness in combating bacterial infections, reducing inflammation, providing antioxidant properties, managing hyperuricemia, and offering renal protection. Notably, clinical trials have confirmed its remarkable efficacy in treating urinary inflammation and stones. We acquired recent research findings concerning CS in the fields of phytochemistry, pharmacology, quality control, and clinical applications via online search. These findings have been summarized and analysed to offer a valuable reference for further comprehensive research, development, and utilization of CS.

## 1 Introduction

Ethnic medicine *Clerodendranthus spicatus* (Thunb.) C.Y.Wu (CS, its Chinese name is “Shencha” or “kidney tea,” syn. *Orthosiphon aristatus* var. *aristatus*, *Orthosiphon spicatus* (Thunb.) Backer, Bakh.f. and Steenis, *Clerodendranthus stamineus* (Benth.) Kudô and *Orthosiphon stamineus* Benth.), a perennial herb of the Lamiaceae family, has been listed in the latest revision of “The Plant List” (http://www.theplantlist.org/) and Medicinal Plant Names Services (http://mpns.kew.org), and commonly known as “cat’s whiskers grass” or “cat’s whiskers tea” (Guo, 2020), with referring as “Yanuomiao” in Dai medicine, is native to the southwest region of Yunnan province in China (Zhao et al., 2004). According to traditional medicinal practices, it is cooling in nature, a mild taste with slight bitterness, and is believed to exhibit properties such as heat-clearing, dehumidifying, stone-expelling, and diuretic effects. As a result, it has gained popularity as a treatment agent for ailments such as cystitis, nephritis, urinary calculus, arteriosclerosis, gout, jaundice, colds for more than two millennia (Hou et al., 2011).

**FIGURE 1:**
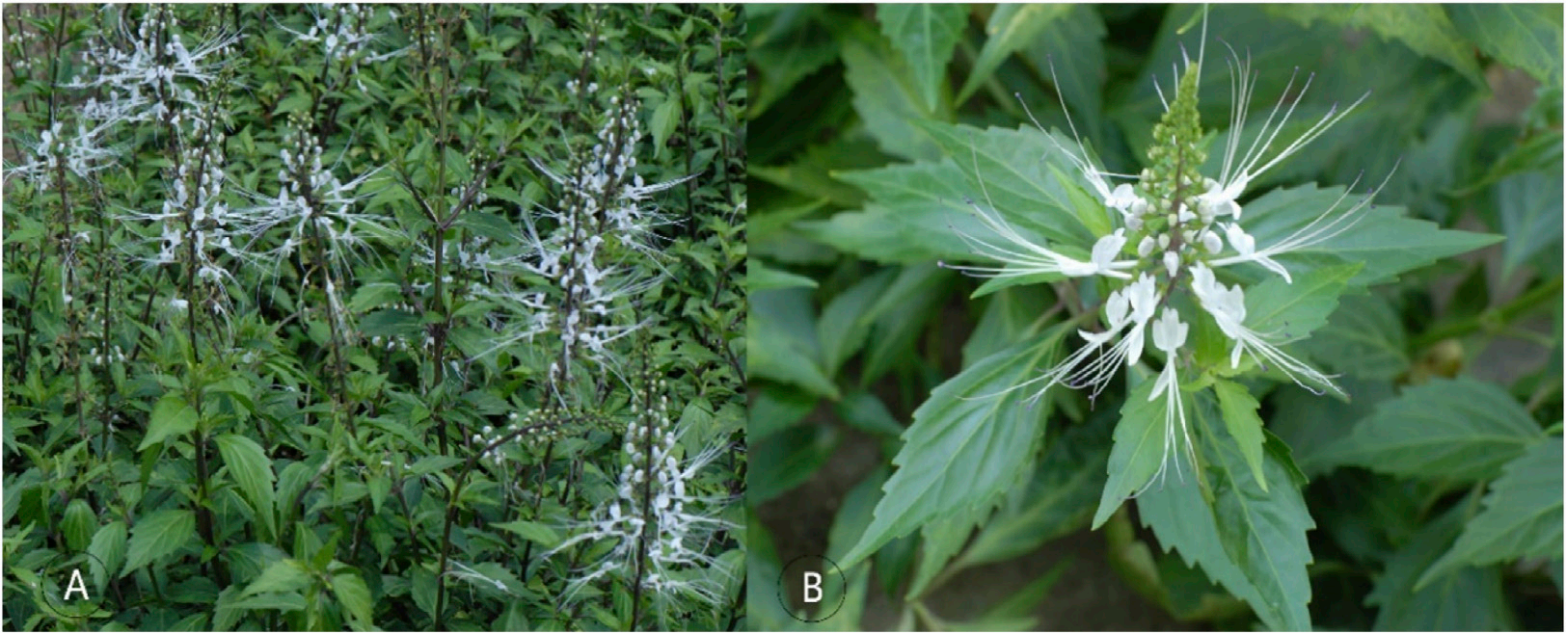
**(A)** Vegetative body **(B)** Blooms (Su et al., 2024).

Contemporary phytochemical analysis has isolated over 300 compounds in CS including flavonoids, terpenoids, phenols, volatile oils, and others (Zhang Y. et al., 2021). Pharmacological investigations confirmed that the plant possesses anti-inflammatory, antibacterial, antioxidant, anti-cancer, anti-gout, and nephroprotective (Hou et al., 2011). The purpose of this review is to provide up-to-date evidence-based information on the phytochemistry, pharmacology, quality control, and clinical application of CS. Therefore, this review summarizes the uniqueness and diversity of the structure of the compounds isolated from this plant, and the latest evidence on the effectiveness of pharmacological effects, and proposes the direction of future in-depth research on this plant as an update and supplement to the information from the previous review (Zhang et al., 2023; Zhang Y. et al., 2021). Therefore, this study provides the latest knowledge and conclusions for an in-depth understanding of this medicinal herb, which may stimulate more comprehensive research, development and utilization. Figure 1 is a representative image of the species CS with its vegetative body and blooms.

## 2 Methodology

The relevant information and data on the phytochemistry, pharmacology, quality control, and clinical application of CS in this review were extracted and collected from published articles in related fields. The chemical structures of the compounds mentioned in the article were prepared using the ChemDraw program, and keywords such as CS, OS, shencha, cat’s whiskers grass, cat’s whiskers tea, research progress, phytochemistry, chemical composition, separation and identification, pharmacological activity, activity quality control, quality standards, and clinical application were used, and Google Scholar, PubMed, and Science Direct were used, Elsevier, Scopus, Embase, Web of Science and China National Knowledge Infrastructure (CNKI), Wanfang, the Chinese Scientific and Technological Periodical Database, the Chinese Biomedical Database (CBM) and other websites for an exhaustive online search. These terms can be used separately or in combination. Then, the retrieved literature was screened and classified according to four aspects: chemical composition, pharmacological effects, quality control and clinical application. The review includes research articles, master’s theses and doctoral dissertations published in English and Chinese, which provide complete information and data.

## 3 Phytochemistry

The research extensively reviewed the literature on the chemical constituents of CS spanning the last 2 decades. To date, over 300 distinct chemical compounds have been isolated, predominantly encompassing flavonoids, terpenoids, anthraquinones, phenylpropanoids, and volatile oils, with flavonoids, phenylpropanoids, and terpenoids possessing the highest content, also serving as the primary active principles within CS.

### 3.1 Flavonoids

**FIGURE 2:**
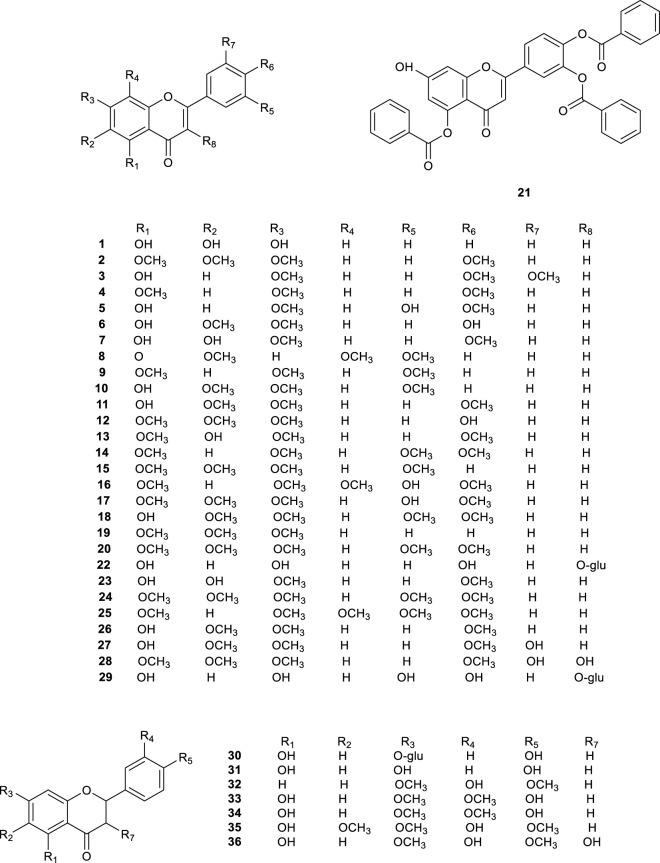
Chemical structures of flavonoids compounds isolated from *Clerodendranthus spicatus*.

Upon extensive exploration of CS, 36 flavonoid derivatives (**1**–**36**, Table 1; Figure 2) have been isolated and identified from the overground part, including 29 flavonoids (**1**–**29**) and seven dihydroflavonoids (**30**–**36**). Notably, Baicalin (**1**) mitigated renal fibrosis via enhancing CPT1α-regulated fatty acid oxidation in diabetic nephropathy (Hu et al., 2024), and flavonoids (**2**, **5**, **7**, **17**, **24**, **27**, and **28**) displayed a diuretic effect by binding to adenosine A_1_ receptor (Yuliana et al., 2009).

**TABLE 1:** List of flavonoids compounds isolated from *Clerodendranthus spicatus*.

No.	Compound name	References
**1**	Baicalin	Sun et al. (2014b)
**2**	Tetramethylscutellarein	Malterud et al. (1989)
**3**	7,3′,4′-Tri-*O*-methylluteolin	Tezuka et al. (2000)
**4**	5,7,4′-Trimethylapigenin	Malterud et al. (1989)
**5**	Pillion	Malterud et al. (1989)
**6**	5,4′-Dihydroxy-6,7-dimethoxy-flavone	Sumaryono et al. (1991)
**7**	5,6-Dihydroxy-7,4′-dimethoxyflavone	Yuliana et al. (2009)
**8**	5-Hydroxy-7,3′,4′-trimethoxy-flavone	Zhao et al. (2004)
**9**	5,7,3′-Trimethoxy-flavone	Malterud et al. (1989)
**10**	5-Hydroxy-6,7,3′-trimethoxy-flavone	Malterud et al. (1989)
**11**	5-Hydroxy-6,7,4′-trimethoxy-flavone	Malterud et al. (1989)
**12**	4′-Hydroxy-5,6,7-trimethoxy-flavone	Sumaryono et al. (1991)
**13**	6-Hydroxy-5,7,4′-trimethoxy-flavone	Sumaryono et al. (1991)
**14**	5,7,3′,4′-Tetramethoxy-flavone	Malterud et al. (1989)
**15**	5,6,7,4′-Tetramethoxy-flavone	Sumaryono et al. (1991)
**16**	3′-Hydroxy-5,7,8,4′-tetramethoxy-flavone	Malterud et al. (1989)
**17**	3′-Hydroxy-5,6,7,4′-tetramethoxy-flavone	Guo (2020)
**18**	5-Hydroxy-6,7,3′,4′-tetramethoxy-flavone	Malterud et al. (1989)
**19**	6,7,8,3′,4′-Pentamethoxy-flavone	Zhu et al. (2021)
**20**	3′,4′,5,6,7-Pentamethoxy-flavanones	Lee et al. (2008)
**21**	3′,4′,5,7-Tetrahydroxy-3′,4′,5-tribenzoate	Guo (2020)
**22**	Astragaloside	Sumaryono et al. (1991)
**23**	Ladanin	Tezuka et al. (2000)
**24**	Sinsensetin	Sumaryono et al. (1991)
**25**	Isohesperetin	Sumaryono et al. (1991)
**26**	Salvigenin	Zhong and Wu (1984)
**27**	Eupatorin	Nagao et al. (2002)
**28**	Eupatorein	Yuliana et al. (2009)
**29**	Isoquercetin	Sumaryono et al. (1991)
**30**	Prunin	Chen et al. (2009)
**31**	(2*S*)-Naringenin	Chen et al. (2009)
**32**	5-Hydroxy-3′,4′,7-trimethoxy-dihydroflavone	Zhang (2019)
**33**	5,4′-Dihydroxy-7,3′-dimethoxy-dihydroflavone	Zhang (2017)
**34**	5,3′-Dihydroxy-7,4′-dimethoxy-dihydroflavone	Zhang (2017)
**35**	5,3′-Dihydroxy-6,7,4′-trimethoxy-dihydroflavone	Zhang (2017)
**36**	3,3′,5-Trihydroxy-4′,7-dimethoxy-dihydroflavone	Zhang et al. (2017)

### 3.2 Terpenoids

CS is rich in terpenoids, predominantly comprising diterpenoids (**46**–**122**) and triterpenoids (**123**–**142**). Recently, researchers have used more advanced extraction and separation techniques to isolate straight chain sesquiterpenoids (**37**–**38**) and norsesquiterpenoids (**39**–**45**).

#### 3.2.1 Sesquiterpenoids

**FIGURE 3:**
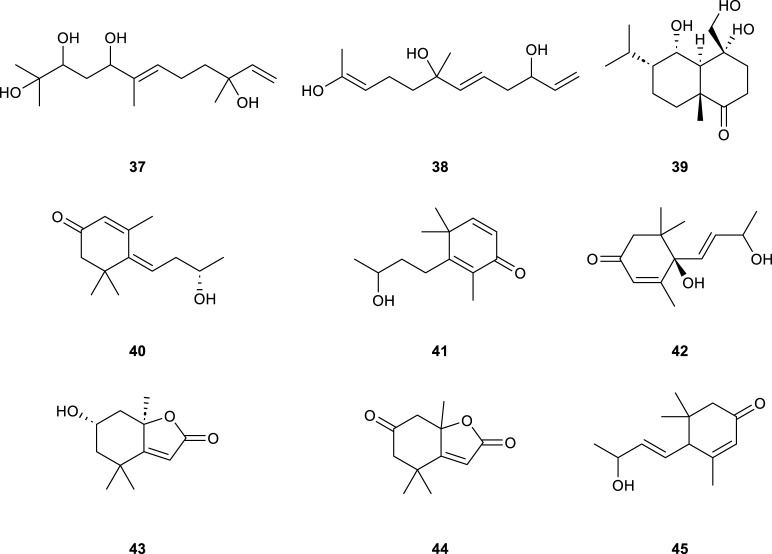
Chemical structures of sesquiterpenoids compounds isolated from *Clerodendranthus spicatus*.

Currently, only nine sesquiterpenoids (Table 2; Figure 3, **37**–**45**) have been isolated from CS. Among them, Loliolide (**43**) can be used as a potential anti-aging bioagent (Zhang et al., 2023), the compounds Loliolide (**43**) and Dehydrololiolide (**44**) have anticholinesterase activities (Xu et al., 2022). In addition, the compound Vomifoliol (**42**) may inhibit the nuclear factor of activated T-cells (NFAT) signaling pathway with calcineurin (CN) as the target enzyme, thereby inhibiting the immune response of Jurkat cells (Zhang X. et al., 2021).

**TABLE 2:** List of sesquiterpenoids compounds isolated from *Clerodendranthus spicatus*.

No.	Type	Compound name	References
**37**	Sesquiterpenoids	2,6,10 Trimethyldodeca-6,11-diene-2,3,5,10-tetraol	Li (2020)
**38**		(2*E*,7*E*)-6,10-Dihydroxy-2,6-dimethyldodeca-2,7,11-trienal	Li (2020)
**39**		6-*epi*-1-oxo-15-Hydroxyverbesindiol	Zhang (2017)
**40**	Norsesquiterpenoids	(4*E*,9*S*)-9-Hydroxy-2,4-megastigmadien-1-one	Luo (2017)
**41**		3-Hydroxybutyl-2,4,4-trimethylcyclohexa-2,5-dienone	Luo (2017)
**42**		Vomifoliol	Luo (2017)
**43**		Loliolide	Li (2020)
**44**		Dehydrololiolide	Li et al. (2017)
**45**		9-Hydroxy-4,7-megastigmadien-3-one	Li et al. (2017)

#### 3.2.2 Diterpenoids

**FIGURE 4:**
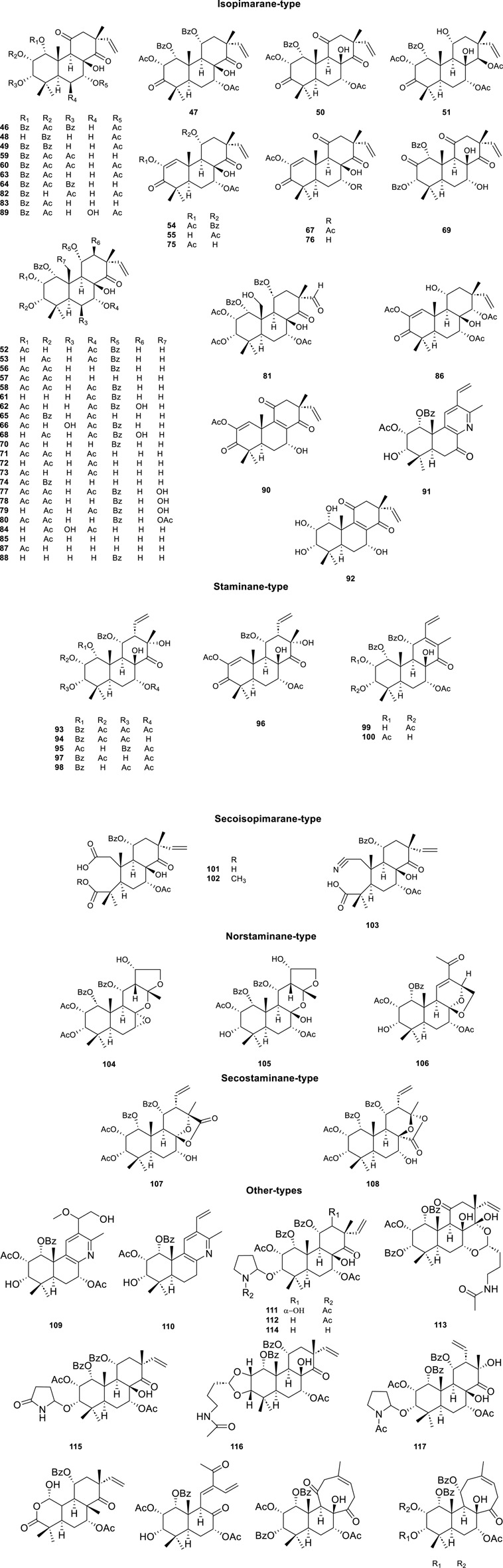
Chemical structures of diterpenoids compounds isolated from *Clerodendranthus spicatus*.

As the characteristic and effective ingredients of CS, a total of 73 diterpenoids (Table 3; Figure 4, **46**–**123**) have been isolated, which were divided into five main categories according to the skeleton type: isopimarane, staminane, secoisopimarane, norstaminane, and secostaminane.

**TABLE 3:** List of diterpenoids compounds isolated from *Clerodendranthus spicatus*.

No.	Type	Compound name	References
**46–51**	Isopimarane	Orthosiphonones A–F	Chen et al. (2020b), Ohashi et al. (2000), Nguyen et al. (2004)
**52–76**		Orthosiphols A, B, D–Z	Takeda et al. (1993), Awale et al. (2003), Awale et al. (2003b), Awale et al. (2001)
**77–81**		Siphonols A–E	Awale et al. (2003a)
**82**		2-*O*-Deacetylorthosiphol J	Awale et al. (2003a)
**83**		3-*O*-Deacetylorthosiphol I	Awale et al. (2003a)
**84**		6-Hydroxyorthosiphol B	Awale et al. (2003a)
**85**		7-*O*-Deacetylorthosiphol B	Awale et al. (2003a)
**86**		14-Deoxo-14-*O*-acetylorthosiphol Y	Nguyen et al. (2004)
**87–91**		Clerodendranthins C–G	Li (2020), Luo et al. (2024)
**92**		Spicatusene A	Luo et al. (2018)
**93–96**	Staminane	Staminols A–D	Nguyen et al. (2004), Awale et al. (2003a)
**97–98**		Neoorthosiphols A, B	Awale et al. (2003a)
**99–100**		Spicatusenes B, C	Luo et al. (2018)
**101–103**	Secoisopimarane	Secoorthosiphols A–C	Awale et al. (2002b)
**104–106**	Norstaminane	Norstaminols A–C	Awale et al. (2003c), Stampoulis et al. (1999)
**107–108**	Secostaminane	Staminolactones A, B	Stampoulis et al. (1999)
**109–117**	Other-types	Clerodendranthins A, B, H–N	Li (2020)
**118**		Nororthosiphonolide A	Awale et al. (2002a)
**119**		Norstaminone A	Awale et al. (2001)
**120–122**		Neoorthosiphonones A–C	Awale et al. (2004), Li et al. (2017)

**FIGURE 5:**
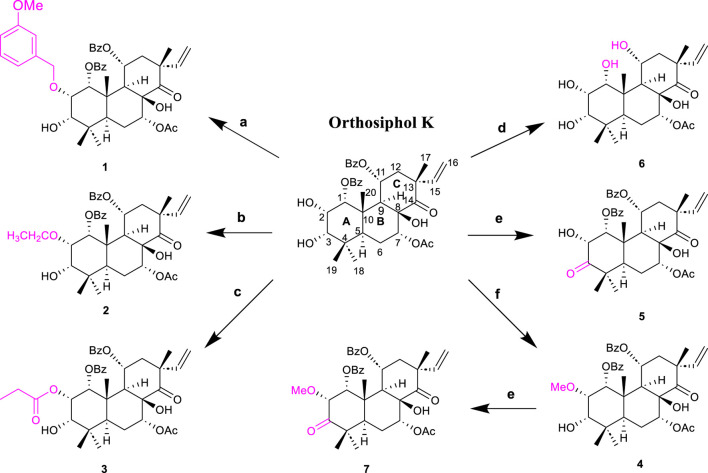
Structures and reactions in modifications of orthosiphol K **(61)**. Reagents and conditions: **(A)** Cs_2_CO_3_, DMF, 3-OMePhCH2Br, 80°C, reflux; **(B)** Cs_2_CO_3_, DMF, CH_3_CH_2_Br, 80°C, reflux; **(C)** Propanoic acid, DMAP, EDCI, CH_2_Cl_2_, 0°C–25°C, 6 h; **(D)** Cs_2_CO_3_, DMF, MeI, r.t., 24 h; **(E)** Jones reagent, acetone, 0°C–25°C, 1.5–2 h; **(F)** NH_4_OH/MeOH, 110°C, reflux, 0.5–1 h (Luo et al., 2024).

The highly oxygenated diterpenoids in CS exhibited various pharmacological properties including anti-inflammation, anti-tumor activity, renal fibrosis prevention, gout management, and diabetes control (Zhang et al., 2023). Highly oxygenated diterpenoids exhibited notable anti-inflammatory activity, specifically the Orthosiphols B, D, and M (**53**, **54** and **63**), as well as Orthosiphonone A and Neoorthosiphol A, surpassing aspirin (Chen et al., 2020a). Orthosiphols A, D (**52**, **54**) and Orthosiphonone A (**46**) modulated gout pathogenesis via the anti-inflammatory and analgesic cascade (Chen et al., 2020b). The compounds Siphonols A–C (**77-79**) and Siphonol E (**81**) showed more potent inhibitory effects on the nitric oxide (NO) production in lipopolysaccharide (LPS)-activated macrophage-like J774.1 cells than a positive control N^G^-monomethyl-_L_-arginine (_L_-NMMA), and Siphonols A–E (**77-81**) represented the first examples of isopimarane-type diterpenes oxygenated at C-20 (Awale et al., 2003). The compounds Spicatusenes B and C (**99** and **100**) exhibited anti-inflammatory properties *in vitro* by inhibiting productions of inflammatory mediators (IL-8, IL-1β, and TNF-α) (Chen et al., 2020a). Additionally, the Spicatusene C (**100**), Orthosiphols K, L, N, R, and W (**61**, **62**, **64**, **68**, **73**), along with Neoorthosiphol A (**97**) and Norstaminol B (**106**) demonstrated antifibrotic effects in TGF-*β*1-induced rat kidneys (Luo et al., 2018). Seven derivatives of orthosiphol K (**61**) were modified by different reagents and conditions (Figure 5). Subsequently, the anti-rheumatoid arthritis activity of these diterpenoid derivatives was evaluated on a TNF-α-induced human rheumatoid fibroblast-like synovial cell model. Of these compounds, compound 2 showed the strongest activity. Based on their inhibitory effect on IL-1b release levels, their structure-activity relationship was preliminarily derived: vinyl-migrated sea pine type diterpenoids showed no activity compared to isopimarane-type diterpenoids. The C-11 OBz group and the C-7 OAc group are important for maintaining activity. For substituents on the ring A, the electron-withdrawing group on the 2-hydroxyl group decreased the activity while the donor group improved the activity. The oxidation of 3-OH to a carbonyl group decreases activity, while Ac substitution on it may increase activity. Thus, the substituents on rings C and A significantly affect the activity of isopimarane-type diterpenoids. The above studies not only enriched the diversity of the structure of diterpenoids, but also showed that isopimarane-type diterpenoids may be a good precursor for the development of anti-rheumatoid arthritis drugs (Luo et al., 2024).

#### 3.2.3 Triterpenoids

**FIGURE 6:**
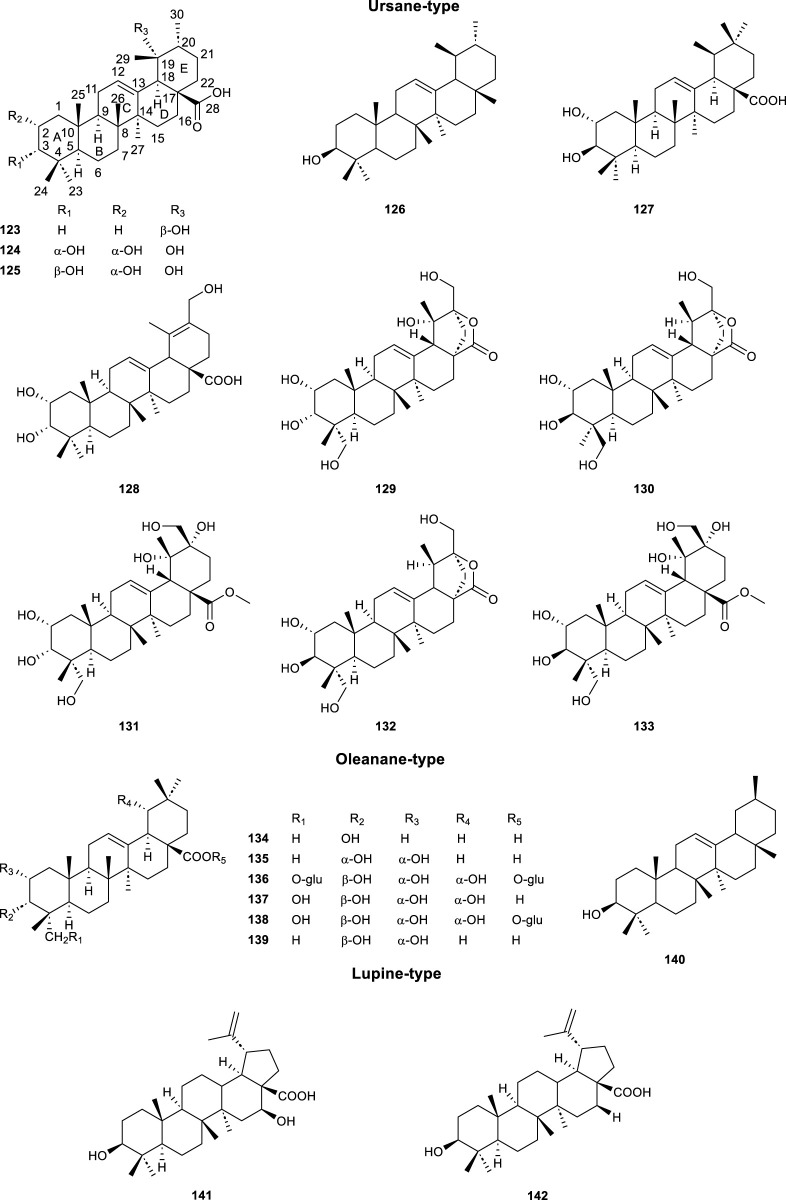
Chemical structures of triterpenoids compounds isolated from *Clerodendranthus spicatus*.

A total of 20 triterpenoids (Table 4; Figure 6, **123**–**142**) was reported from CS. One of the representatives of pentacyclic triterpenoids was Ursolic acid (UA, **123**), which possessed important biological effects, including anti-inflammatory, anticancer, antidiabetic, antioxidant and antibacterial effects (Mlala et al., 2019). It has been reported that the C-3 (hydroxyl), C12-C13 (double bond) and C-28 (carboxylic acid) positions of UA have been modified (such as acetylation, methylation, and esterification) to obtain several ursolic acid derivatives, which have stronger potency, bioavailability and water solubility than UA (Mlala et al., 2019). The α-Amyrin (**126**) demonstrated superior efficacy in stimulating uric acid excretion compared to benzbromarone (Chen et al., 2020b). Maslinic acid (**139**) was one of the representatives of oleanane-type triterpenoids, which has a variety of biological activities, such as anti-tumor, hypoglycemic, anti-inflammatory, and anti-parasitic (Yan et al., 2024). β-Amyrin (**140**) was displayed important anti-Toxoplasma activity (Cardona-Trujillo et al., 2024). Something special, Betulinic acid (**141**) was a triterpene natural product which has shown antiparasitic activity against *Leishmania*, *Trypanosoma cruzi*, and *Plasmodium* (Rocha et al., 2022).

**TABLE 4:** List of triterpenoids compounds isolated from *Clerodendranthus spicatus*.

No.	Type	Compound name	References
**123**	Ursane	Ursolic acid	Tan et al. (2009)
**124**		Euscaphic acid	Tan et al. (2009)
**125**		Tormentic acid	Tan et al. (2009)
**126**		*α*-Amyrin	Chen et al. (2020b)
**127**		2*α*-Hydroxy-ursolic acid	Wang et al. (2016)
**128–132**		Spicatusoids A–E	Luo et al. (2017)
**133**		Vitexnegheterion H	Luo et al. (2017)
**134**	Oleanane	Oleanolic acid	Tan et al. (2009)
**135**		2*α*,3*α*-Dihydroxyolean-12-en-28-oic acid	Tan et al. (2009)
**136**		Arjungenin-23,28-*bis*-*O*-glucopyranoside	Chen et al. (2009)
**137**		Arjunolic acid	Luo et al. (2018)
**138**		Arjung lucoside I	Chen et al. (2009)
**139**		Maslinic acid	Tan et al. (2009)
**140**		*β*-Amyrin	Chen et al. (2020b)
**141**	Lupine	Betulinic acid	Chen et al. (2020b)
**142**		Orthosiphonoic acid	Hossain and Ismail (2005)

### 3.3 Phenylpropanoids

**FIGURE 7:**
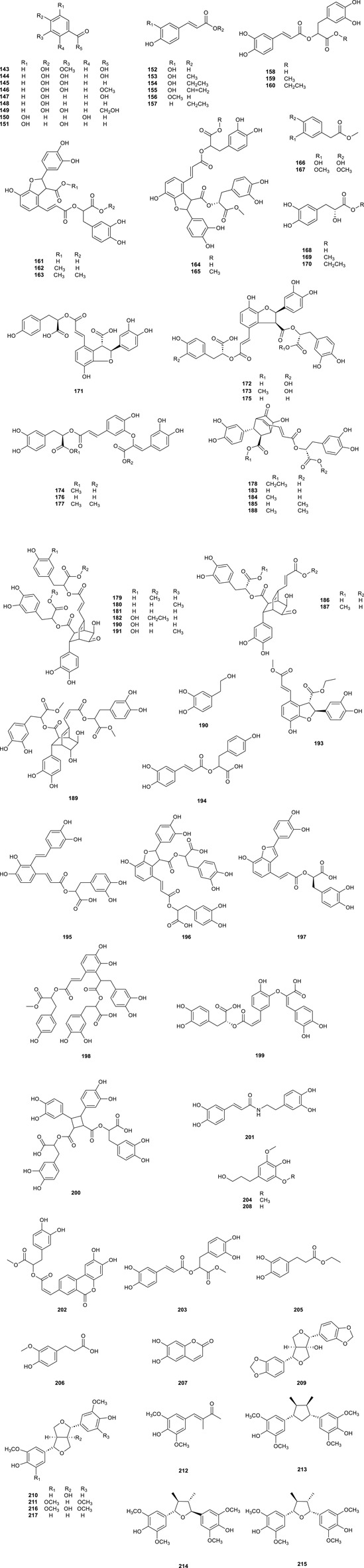
Chemical structures of phenylpropanoids compounds isolated from *Clerodendranthus spicatus*.

Phenylpropanoids, which were characteristic constituents in CS, and exhibited numerous therapeutic properties, such as anticancer, antioxidant, anti-inflammatory, and neuroprotective effects, with 75 phenylpropanoids (Table 5; Figure 7, 143–217) was isolated from CS. Analysis via HPLC-ESI-MS showed the presence of compounds such as Caffeic acid (**152**), Ferulic acid (**156**), *p*-Hydroxybenzoic acid (**147**), Rosmarinic acid (**158**), Danshensu (**168**), and Protocatechuic acid (**144**) in aqueous extracts of CS, which delayed exogenous senescence by defending antioxidant activities and suppressing inflammation (Wang et al., 2016). Rosmarinic acid (**158**) was found as a major component responsible for the antioxidant activity in CS extract (Nuengchamnong et al., 2011), and Orthosiphoic acid B (**172**) and C (**173**) displayed modest anti-HIV-1 protease activity (Sun et al., 2014). Clerodens A**–**J (**178**–**187**), which with a distinctive bicycle [2.2.2] octane moiety, and Cleroden D (**181**) notably inhibited LPS-induced NO production in RAW 264.7 (Ma et al., 2015), while the Cleroden E (**182**) showed notable antibacterial potency to drug-resistant bacterial properties *in vitro*. Additionally, Helisterculin C (**190**), featuring a rare bicyclol [2.2.2] octane moiety previously isolated phenolic acid derivatives, Helisterculins C (**190**) and D (**191**), exhibited moderate neuroprotective activity (Zhou et al., 2017).

**TABLE 5:** List of phenylpropanoids compounds isolated from *Clerodendranthus spicatus.*

No.	Compound name	References
**143**	Vanillic acid	Chen et al. (2017)
**144**	Protocatechuic acid	Wang et al. (2016)
**145**	Protocatechualdehyde	Chen et al. (2017)
**146**	Protocatechuic acid methyl ester	Fan et al. (2013)
**147**	*p*-Hydroxy-benzoic acid	Wang et al. (2016)
**148**	*p*-Hydroxy-benzaldehyde	Chen et al. (2017)
**149**	3,4-Dihydroxyphenacyl alcohol	Zhao et al. (2004)
**150**	2,5-Hydroxy-benzaldehyde	Fan et al. (2013)
**151**	3,5-Dihydroxybenzaldehyde	Chen et al. (2017)
**152**	Caffeic acid	Wang et al. (2016)
**153**	Methyl caffeate	Sun et al. (2014a)
**154**	Ethyl caffeate	Sun et al. (2014a)
**155**	Vinyl acetate	Luo et al. (2018)
**156**	Ferulic acid	Wang et al. (2016)
**157**	Ethyl coumarate	Luo et al. (2018)
**158**	Rosmarinic acid	Wang et al. (2016)
**159**	Methyl rosmarinate	Wang et al. (2016)
**160**	Ethyl rosmarinate	Wang et al. (2016)
**161**	Lithospermic acid	Chen et al. (2017)
**162**	Methyl lithospermate	Chen et al. (2017)
**163**	Dimethy lithospermate	Chen et al. (2017)
**164**	Methyl lithosperma B	Wang et al. (2007)
**165**	Dimethyl lithospermate B	Wang et al. (2007)
**166**	3,4-Dihydroxyphenyllactate	Wang et al. (2016)
**167**	3,4-Methoxy benzoethylic methyl ester	Fan et al. (2013)
**168**	Danshensu	Sun et al. (2014a)
**169**	Danshensu methyl ester	Chen et al. (2017)
**170**	Ethyl 3,4-dihydroxyphenyllactate	Sun et al. (2014a)
**171–175**	Orthosiphoic acids A–E	Sun et al. (2014a), Wang et al. (2023)
**176**	3′-*O*-(8″-Z-caffeoyl) Rosmarinic acid	Sun et al. (2014a)
**177**	Clerodendranoic acid	Zheng et al. (2012)
**178–187**	Clerodens A–J	Ma et al. (2015), Li et al. (2016)
**188–191**	Helisterculins A–D	Zhou et al. (2017), Chen et al. (2017)
**192**	3,4-Dihydroxyphenylethanol	Ma et al. (2015), Li et al. (2016)
**193**	(7′*S*,8′*S*)-8-Epiblechnic acid diacetate	Li et al. (2017)
**194**	Isorinic acid	Sun et al. (2014a)
**195–199**	Salvianolic acids A–C,E,H	Sun et al. (2014a), Chen et al. (2009)
**200**	Sagerinic acid	Nuengchamnong et al. (2011)
**201**	*N*-(*E*)-Cafeoyldopamine	Zhu et al. (2021)
**202**	Logan-1	Luo et al. (2018)
**203**	(±)-Rosmarinsauremethylester	Luo et al. (2018)
**204**	Dihydrosinapyl alcohol	Sun et al. (2014a)
**205**	Ethyl dihydrocaffeate	Chen et al. (2017)
**206**	Dihydroferulic acid	Chen et al. (2017)
**207**	Esculetin	Zhao et al. (2004)
**208**	Dihydroconiferol	Sun et al. (2014a)
**209**	Paulownin	Li (2020)
**210**	8-Hydroxypinoresinol	Chen et al. (2009)
**211**	Syringaresinol	Chen et al. (2009)
**212**	Sacidumol A	Li et al. (2017)
**213–215**	Fragransins B_1_–B_3_	Li et al. (2017)
**216**	1-Hydroxysyringaresinol	Chen et al. (2009)
**217**	(**+**)-Pinoresinol	Zhang et al. (2017)

### 3.4 Volatile oil

**FIGURE 8:**
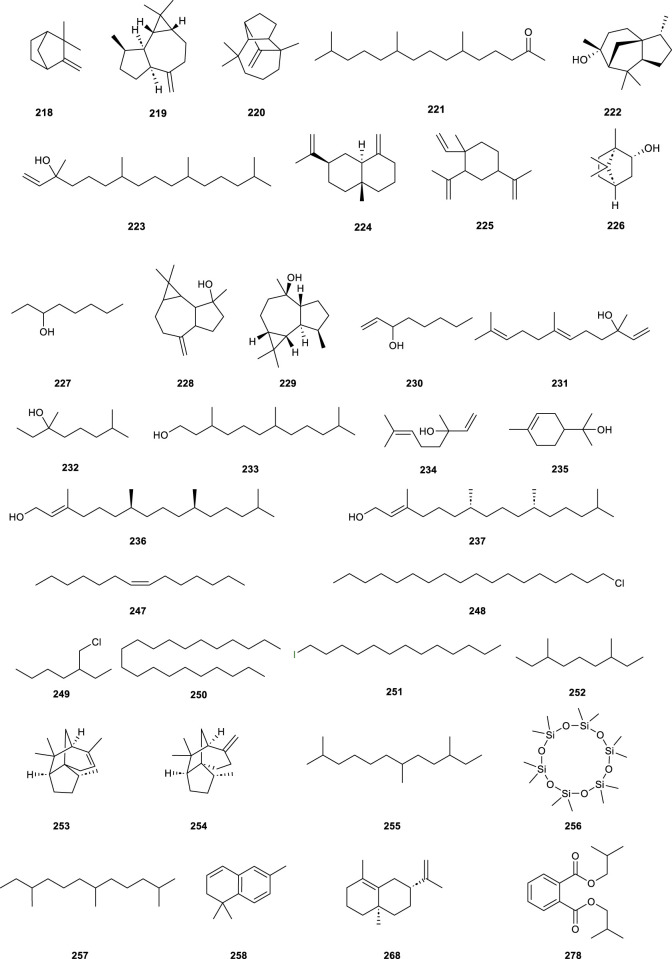
Chemical structures of partially volatile oil compounds isolated from *Clerodendranthus spicatus*.

CS is abundant in volatile oil constituents, yet there are few studies on their pharmacological activity and medical application, predominantly highlighting chemical composition identification and content determination. 81 chemical constituents were identified (Table 6; Figure 8, **218–298**) in volatile oils extracted via conventional steam distillation and solvent extraction coupled with gas chromatography–mass spectrometry (GC-MS), primarily comprising alcohols, alkenes, ketones, naphthalene, esters, and alkanes. Cedrol (**222**) as the most abundant sesquiterpene (Li et al., 2022), comprising 53.64%, which exhibiting antibacterial activity (Ma, 2013). According to the literature, Cedrol (**222**) has antibacterial activity (Chang et al., 2023).

**TABLE 6:** List of volatile oil compounds isolated from *Clerodendranthus spicatus*.

No.	Name	Molecular formula	Relative molecular mass	Relative quantity (%)
Monoterpenoids				
**218**	Camphene	C_16_H_16_	136	0.2
Phenylpropanoids				
**219**	Aromadendrene	C_15_H_24_	204	1.12
**220**	Longifolene	C_15_H_24_	204	0.73
**221**	6,10,14-Trimethyl-2-pentadecanone	C_18_H_36_O	268	0.47
**222**	Cedrol	C_15_H_26_O	222	53.64
Diterpenoids				
**223**	Isophytol	C_20_H_40_O	296	0.09
Alcohols				
**224**	β-Selinene	C_15_H_24_	204	0.61
**225**	β-Elemene	C_15_H_24_	204	0.48
**226**	Borneol	C_10_H_18_O	154	0.33
**227**	3-Octanol	C_8_H_18_O	130	0.13
**228**	Spathalenol	C_15_H_24_O	220	4.13
**229**	Gobulol	C_15_H_26_O	222	3.24
**230**	1-Octen-3-ol	C_8_H_16_O	128	3.92
**231**	Trans-nerolidol	C_15_H_26_O	222	0.16
**232**	3,7-Dimethyloctan-3-ol	C_10_H_22_O	158	0.05
**233**	Hexahydrofarnesol	C_15_H_32_O	228	0.24
**234**	Linalool	C_10_H_18_O	154	0.13
**235**	*p*-Menth-1-en-8-ol	C_10_H_18_O	154	0.56
**236**	3,7,11,15-Tetramethyl-2-hexadecen-1-ol	C_20_H_40_O	296	0.38
**237**	Phytol	C_20_H_40_O	296	0.39
**238**	3,7-Dimethyl-1,5,7-octatrien-3-ol	C_10_H_16_O	152	0.92
**239**	*cis*-1,2-Cyclododecanediol	C_12_H_24_O_2_	200	0.30
**240**	4-Methyl-1-(1-methylethyl)-3-cyclohexen-1-ol	C_10_H_18_O	154	0.12
**241**	*cis*-1-Methyl-2-(1-methylethyl)-cyclobutaneethanol	C_10_H_18_O	154	1.48
**242**	1,1,4,7-Tetramethyldecaoxy-1H-cyclopropane[e]fur-4-ol	C_15_H_26_O	222	1.17
**243**	(1.a.,3.a.,4.β.,6.a.)-4,7,7-Trimethyl-dicyclo[4.1.0]heptane-3-ol	C_10_H_18_O	154	0.06
**244**	4,4,11,11-Tetramethyl-7-tetracyclo[6.2.1.0(3.8)0(3.9)]-11-ol	C_15_H_24_O	220	0.10
**245**	1,5,9-Trimethyl-12-(1-methylethyl)-4,8,13-cyclotetradecen-1,3-diol	C_20_H_36_O_2_	308	0.08
**246**	1,2,3,4,4a,8a-Hexahydro-.a.,.a.,4a,8-tetramethyl-,[2R-(2.a.,4a.a.,8a.a.)]-2-naphthalenemethanol	C_15_H_24_O	220	0.70
Hydrocarbons				
**247**	7-Tetradecene	C_14_H_28_	196	0.15
**248**	1-Chlorooctadecane	C_18_H_37_Cl	288	0.49
**249**	2-Ethylhexyl chloride	C_8_H_17_Cl	148	0.08
**250**	Heneicosane	C_21_H_44_	296	0.23
**251**	1-Iodotridecane	C_13_H_27_	310	0.10
**252**	3,7-Dimethylnonane	C_11_H_24_	156	0.02
**253**	*α*-Cedrene	C_15_H_24_	204	3.53
**254**	β-Cedrene	C_15_H_24_	204	2.51
**255**	2,7,10-Trimethyldodecane	C_15_H_32_	212	0.09
**256**	Dodecamethylcyclohexasiloxane	C_12_H_36_O_6_Si_6_	444	0.04
**257**	2,6,10-Trimethyldodecane	C_15_H_32_	212	0.05
**258**	1,2-Hydro-1,1,6-trimethylnaphthalene	C_13_H_16_	172	0.06
**259**	8,9-Deoxy-cycloisolongifolene	C_15_H_22_	202	0.27
**260**	8-Hydro-13-ol- cedrene	C_15_H_24_O	220	0.32
**261**	3-Eicosane	C_20_H_38_	278	0.07
**262**	(+)10-(Acetylmethyl)-3-Naphene	C_13_H_20_O	192	0.6
**263**	1,6-Dimethyl-4-(1-methylethyl)-naphthalene	C_15_H_18_	198	0.24
**264**	2,6-Dimethyl-1,6-diol-2,7-octadiene	C_10_H_18_O_2_	170	0.38
**265**	1-(1,5-Dimethyl-4-hexenyl)-4-methylbenzene	C_15_H_22_	202	0.39
**266**	1,2,3,6-Tetramethyl-dicyclo[2.2.2]octa-2-ene	C_12_H_2_O	164	0.05
**267**	1,7,7-Trimethyl-2-vinylbicyclo[2.2.1]hept-2-ene	C_12_H_18_	162	0.06
**268**	4a,8-Dimethyl-2-propene-1,2,3,4,4a,5,6,7-octahydronaphthalene	C_15_H_24_	204	0.18
**269**	2-Methyl-5-(1-methylethyl)-bicyclo[3.1.0]hexahepten-2-ene	C_15_H_24_	204	0.30
**270**	4-Methylene-2,8,8-trimethyl-2-ethylene-dicyclo[5.2.0]nonane	C_15_H_24_	204	0.47
**271**	(*Z*,*Z*,*Z*)-2,3-Dihydroxypropyl ester-9,12,15-Octadecatrienoic acid	C_21_H_36_O_4_	352	0.11
**272**	1-Isopropyl-4,7-dimethyl-1,3,4,5,6,8a-hexahydro-4a(2H)-naphthol	C_15_H_26_O	222	0.89
**273**	(*E*,*E*)-1,5-Dimethyl-8-(1-methylacetal)-1,5-cyclodecadiene	C_15_H_24_	204	1.20
**274**	7-Isopropyl-1,4a-dimethyl-1,2,3,4,4a,5,6,8a-octahydro-1-naphthol	C_15_H_26_O	222	0.82
**275**	2-Methylene-5-(1-methylvinyl)-8-methyl-dicyclo[5.3.0]decane	C_15_H_24_	204	0.30
**276**	7,7-Dimethyl-5-isopropyl-2-isopropenyl-bicyclo[4.1.0]-3-heptene	C_15_H_24_	204	0.06
**277**	1-Vinyl-1-methyl-2-(1-methylethyl)-4-(1-methylacetal)-cyclohexane	C_15_H_24_	204	0.46
Esters				
**278**	Diisobutyl phthalate	C_16_H_22_O_4_	278	0.11
**279**	Methyl 6,9,12,15-docosatetraenoate	C_23_H_38_O_2_	346	0.30
Ketones				
**280**	5-Methyl-5-isopropyl-3-heptyn-2,6-dione	C_14_H_20_O_3_	236	0.21
**281**	1-Cyclopropyl-1-dodecanyl methylketone	C_15_H_28_O	224	0.03
**282**	3-Isopropylidene-5-methyl-hexa-4-en-2-one	C_10_H_16_O	152	0.02
**283**	6-Vinyl-2,2,6-trimethyl-2H-pyran-3(4H)-one	C_10_H_16_O_2_	168	1.84
**284**	1,1,4a-Trimethyl-3,4,4a,5,6,7-hexahydro-2(1H)-naphthyone	C_13_H_20_O	192	0.47
**285**	1-(2,6,6-Trimethyl-1,3-cyclohexadien-1-yl)-2-buten-1-one	C_13_H_18_O	190	1.02
**286**	4-(2,4,4-Trimethylcyclohexane-1,5-diethyl)-3-buten-2-one	C_13_H_18_O	190	0.18
**287**	4-(2,2-Dimethyl-6-methylenecyclohexylethylene)-3-methylbutane-2-one	C_14_H_22_O	206	0.09
Others				
**288**	Epoxyisociferene	C_15_H_24_O	220	0.11
**289**	*α*-Copaene-11-ol	C_15_H_24_O	220	0.12
**290**	1,8-Cyclotetramethylene	C_14_H_20_	188	0.18
**291**	Serinene(C_15_H_26_O)	C_15_H_26_O	222	1.01
**292**	Serinene(C_15_H_24_)	C_15_H_24_	204	0.17
**293**	Cadinene	C_15_H_24_	204	0.39
**294**	*α*-Cadinol	C_15_H_26_O	222	0.50
**295**	1-Methyl-3-(1,3,3-trimethyl-dicyclo[4.1.0]heptan-2-yl)-propenyl acetic acid	C_16_H_26_O_2_	250	0.04
**296**	[1aS-(1a.a.,3a.a.,7a.β.,7b.a.)]-Hydrogen decahydrate-1,1,3a-trimethyl-7-methylene-1H-cyclopropane[a]naphthalene	C_15_H_24_	204	0.04
**297**	[1aR-(1a.a.,7.a.,7a.β.,7b.a.)]-1a,2,3,5,6,7,7a,7b-Octahydro-1,1,4,7-tetramethyl-1H-cyclopropene[e]O	C_15_H_24_	204	0.94
**298**	[1aR-(1a.a.,4.a.,4a.β.,7b.a.)]-1a,2,3,4,4a,5,6,7b-Octahydro-1,1,4,7-tetramethyl-1H-cyclopropenyl[e]-austroce	C_15_H_24_	204	0.28

### 3.5 Others

**FIGURE 9:**
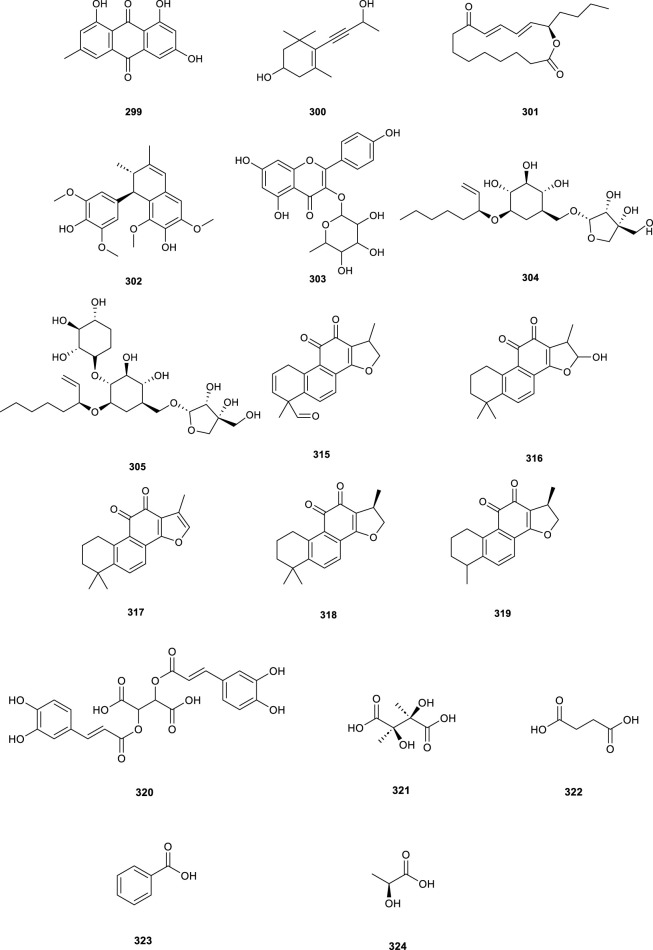
Chemical structures of other compounds isolated from *Clerodendranthus spicatus*.

Additional compounds isolated from CS include anthraquinones, cyanosides, alkaloids, organic acids, and diverse types of components (Table 7; Figure 9, **299–324**). Notably, amides compounds (**311**–**313**) demonstrated anti-inflammatory properties, confirming their role as major anti-inflammatory constituents (Li et al., 2022).

**TABLE 7:** List of Other compounds isolated from *Clerodendranthus spicatus*.

No.	Type	Name	References
**299**	Anthraquinones	Emodin	Chen et al. (2009)
**300**	Terpene alcohols	3-Hydroxy-7,8-dehydro-*β*-ionol	Li et al. (2017)
**301**	Fatty acids	Axillactone B	Li (2020)
**302**	Phenolic acids	trans-7,8-Dihydro-(4′-hydroxy-3′5′-dimethoxyphenyl)-1,3-dimethoxy-6,7-dimethyl-2-naphthol	Luo (2017)
**303**		Kaempferol 3-*O*-*α*-L-rhamnopyranoside	Luo et al. (2018)
**304–305**	Alkyl glycosides	Clerspides A, B	Zou et al. (2008)
**306**	Sterols	β-Sitosterol	Zhong and Wu (1984)
**307**		Caroteneside	Zhong and Wu (1984)
**308**	Terpeneones	3-Hydroxy-4-oxo-7,8-dihydro-β-ionone	Li et al. (2017)
**309**		3-Hydroxy-5,6-epoxy-*β*-ionone	Li et al. (2017)
**310**		6-Hydroxy-1-oxo-7,8-dihydro-*β*-ionone	Luo et al. (2018)
**311**	Amides	*N*-*cis*-Ferulotyromine	Li et al. (2022)
**312**		*N*-*trans*-Ferulyltyramine	Li et al. (2022)
**313**		*trans*-*N*-Cinnamoyltyramine	Li et al. (2022)
**314**	Tanshinones	Neoechinulin A	Li et al. (2022)
**315**		Tanshinoldehyde	Guo (2020)
**316**		Tanshinone A	Guo (2020)
**317**		Tanshinone IIA	Guo (2020)
**318**		Cryptotanshinone	Guo (2020)
**319**		15,16-Dihydrotanshinone	Guo (2020)
**320**	Organic acids	Cichoric acid	Guo (2020)
**321**		Tartaric acid	Li et al. (2002)
**322**		Succinic Acid	Li et al. (2002)
**323**		Benzoic acid	Li et al. (2002)
**324**		Lactic acid	Li et al. (2002)

## 4 Pharmacology

Previously documented studies indicate CS possesses diverse pharmacological activities such as anti-inflammatory, antibacterial, antioxidant, anti-neoplastic, anti-gout, and renal protection properties. Notably, it is frequently utilized for treating nephritis, gout, urinary tract infections, kidney stones, and related conditions. An in-depth analysis on these pharmacological actions was thus discussed subsequently.

### 4.1 Anti-inflammatory

Thousands of years of ethnic medicine practice has proved that CS has superior efficacy in the treatment of urinary system inflammation, and modern phytochemical and biological activity research has unearthed the material basis for CS to exert anti-inflammatory effects. The hyperoxy diterpenoids derived from ethanolic extract of CS exhibited anti-inflammatory potency superior to aspirin *in vitro* by inhibiting the pro-inflammatory factors TNF-α, IL-1β, and IL-8 stimulated via LPS in renal epithelial cells (HK-2 cells) (Chen et al., 2020a). In addition, phenolic acid, the Clerodens A–D (**178–181**), with a dicyclic [2.2.2] octene framework, was isolated from the extract CS demonstrating the anti-inflammatory activity by suppressing NO produced by RAW 264.7 cells induced by LPS (Ma et al., 2015) The total flavonoids of CS proved effective against bacterial prostatitis in rats, potentially related to inhibiting TNF-α and IL-8 inflammation and reducing oxidative stress (Chen and Yang, 2016). Three identified amide compounds, *N*-*cis*-Feruloyltyramine (**312**), *N*-*trans*-Feruloyltyramine (**313**), and *trans*-*N*-Cinnam-oyltyramine (**314**), isolated from the middle and low polarity parts of CS, which exhibited potent anti-inflammatory activity in the LPS-stimulated RAW 264.7 macrophage inflammation model. The results of this study support the idea that amide compounds were one of the main components of the anti-inflammatory effect of CS (Li et al., 2022).

### 4.2 Antibacterial

Nowadays, bacterial infections have increased all over the world and antibiotics resistance has emerged as a challenging healthcare issue, therefore screening of medicinal plants to explore new antibacterial agents has become a top priority (Alshawsh et al., 2012). The aqueous extract of CS suppresses the expansion of *Gram-negative bacilli* and *Staphylococci* such as *Escherichia coli* and *Klebsiella pneumonia* (Yi et al., 2013). Cleroden E (**182**) with a bicyclic [2.2.2] octene functional showed moderate antibacterial activity against various clinically isolated drug-resistant strains such as *Staphylococcus aureus* ATCC 33591 (MRSA), *Staphylococcus epidermidis* 09-3 (MRSE), *Enterococcus faecalis* ATCC 51299 VRE, *E. coli* 09-1 ESBLs, and *Klebsiella pneumoniae* ATCC BAA-2146 NDM-1, exhibiting minimum inhibitory concentrations of 3.2, 11.8, 3.2, 6.4, and 3.2 *μg*/mL *in vitro* (Li et al., 2016). In conclusion, this plant exhibited promising antibacterial properties, particularly against *Gram-positive bacteria*. Both past medicinal practices and modern scientific research have highlighted the potential of CS as an antimicrobial agent.

### 4.3 Antioxidant

The physiological and pathological processes of many diseases are accompanied by free radical-mediated lipid peroxidation, such as arteriosclerosis, heart diseases, aging process and cancer. The petroleum ether (PE), chloroform (CE), ethyl acetate (EAE), and water (WE) extracts of CS demonstrated DPPH, O^2−^, ⋅OH, Fe^2+^, and ROS scavenging activities *in vitro*. Significantly, EAE and WE extract exhibited potency to decrease MDA levels and enhance GSH-Px levels in renal homogenates, concurrently suppressing mitochondrial swelling under concentration-dependent conditions (Chen et al., 2014). Besides, research noted that the methanol extract of CS exhibits strong scavenging capabilities for superoxide ions and DPPH free radicals *in vitro*, attributing its efficacy to Sinsensetin (**24**) and 3′-Hydroxy-5,6,7,4′-tetramethoxyflavone (**17**) (Yam et al., 2007). Other studies showed that there were significant correlations between phenolic compounds and antioxidant properties of medicinal plants. The ethyl acetate fraction (EAF) of aqueous-methanolic extract of CS, which had the highest total phenol content and high total flavonoid content, and the EAF fraction showed excellent antioxidant activity in a number of subsequent antioxidant tests such as *β*-carotene bleaching (BCB) and DPPH free radical determination (Abdelwahab et al., 2011). Even more surprisingly, a number of phenolic compounds that exert antioxidant effects were identified in another study, such as Helisterculin C (**190**), Salvianolic acid B (**196**), Orthosiphoic acid E (**175**) and Ethyl caffeate (**154**) might be the main antioxidant constituents (Wang et al., 2023). These findings supported the idea that the antioxidant activity of phenolic compounds was found to be mainly due to their scavenging and redox properties, through neutralizing and quenching free radicals (Alshawsh et al., 2012). It was envisaged that phenolic compounds in CS will be used as an antioxidant to support the treatment of many major diseases in the future.

The randomized trial in a mouse model of UV-induced skin damage demonstrated that the WE extract of CS successfully reduced the levels of inflammatory cytokines such as IL-1β, IL-6, TNF-α, COX-2, and PGE2, rejuvenated collagen density, and restricted on production of matrix metalloproteinases (Wang et al., 2016), suggesting that WE with ability to postpone exogenously induced aging via antioxidant and anti-inflammatory properties.

### 4.4 Anti-tumor

Tumor/cancer is a prominent cause of mortality in humans, and numerous studies have proposed that naturally occurring compounds derived from plants could serve as potential agents for combating tumors (Xu et al., 2015). Recent research on the anti-tumour effects of CS has mainly focused on non-small cell lung, colon and liver cancers Studies *in vivo* and *in vitro* have proved that CS has an anti-tumor effect. Investigations into the *in-vitro* anti-tumor effects of CS demonstrated that EAE extract potent inhibitory activity against human non-small cell lung cancer cells A549, human ileocecal cancer cells HCT-8, and human hepatocellular carcinoma cells BEL-7402, exhibiting IC_50_ values of 194.61, 283.05, and 238.61 μg/mL (Zheng et al., 2019). Furthermore, extract (C5EOSEW5050ESA) (400 mg/kg) of CS potently suppresses the Notch signaling cascade by diminishing Notch1 ICD, HES-1, and HEY-2 signalings, thereby impeding Notch signaling function, minimizing expression of multidrug resistance genes, and epithelial-mesenchymal transition markers in gemcitabine-resistant cells, potentially auguring as a complementary treatment agent for drug-resistant pancreatic cancer (Yehya et al., 2022).

*In-vivo* experiments, the 50% ethanol extract (EE) of CS at a dose of 200 mg/kg resulted in an 83.39% ± 4.1% reduction in colorectal tumor proliferation in athymic mice. In-depth mechanistic studies have convinced that CS exerts the antiangiogenic effect by blocking VEGF signaling pathway, and eventually the antiangiogenic property could be the principle factor for the potent antitumor property of CS, and the antiangiogenic effect may be due to the collective contribution of phytochemicals particularly, Rosmarinic acid (**158**), Eupatorine (**27**), Sinensetin (**24**), Betulinic acid (**141**), and 3′-Hydroxy-5,6,7,4′-tetramethoxyflavone (**17**) in CS (Ahamed et al., 2012).

### 4.5 Anti-hyperuricemia and anti-gout

Many studies have shown that hyperuricemia (HUA) and gout are independent risk factors for chronic kidney disease (CKD), obesity, hypertension, type 2 diabetes, cardiovascular and cerebrovascular diseases, and are independent predictors of premature mortality (Bardin and Richette, 2017). CS has been extensively utilized the treatment of HUA and gout in folk and clinical practice, demonstrating notable effectiveness. Ethyl acetate extract (OSE) of CS that was shown to have curative effects in a mouse model of potassium oxazinate (PO) induced HUA after treatment at a dose of 500–2,000 mg/kg for 7 days. It is hypothesized that its impact may be achieved by decreasing xanthine oxidase (XOD) and adenosine deaminase (ADA) activity in the liver, downregulating the mRNA and protein expression levels of uric acid transporters URAT 1 and GLUT 9 and upregulating OAT 1 and OAT 3 in the renal. Subsequently, eight components were identified from the OSE extracts by UPLC/ESI-MS, including Protocatechualdehyde (**145**), Caffeic acid (**152**), Rosmarinic acid (**158**), Caffeic acid tetramer, Lithospermic acid (**161**), Salvianolic acid B (**196**), isorhamnetin-3-O-hexoside and caffeic acid derivative (Xu et al., 2020). Furthermore, the anti-HUA activity of Orthosiphols A, B, N, and α-Amyrin (**52**, **53**, **64** and **126**) isolated from the EAE extract of CS was superior to that of benbromarone, and most diterpenes with high oxygen content have significant anti-gout arthritis and analgesic activity, the results exhibited that CS modulated pathological state of gout mainly via the anti-inflammatory and analgesic cascade. (Chen et al., 2020b). Similarly, WE extracts of different doses of CS have exhibited significant anti-hyperuricemia and anti-gout activities through diverse pharmacodynamic mechanisms, such as: 1. CS improved the pathological state of HUA by regulating the structure of intestinal flora (increased the abundance of *Roseburia* and *Enterorhabdus*, and decreased the abundance of *Ileibacterium* and *UBA1819*) and remodeling metabolic disorders (returned the levels of differential metabolite to normal) (Chen et al., 2023); 2. CS exerted anti-HUA activity via changing the composition of intestinal microbiota (reduced the abundance of *unidentified-Ruminococcaceae* and *Lachnospiraceae-NK4A136-group*), metabolites (increased 17 metabolites such as lactose, 4-oxovaleric acid, butyric acid, etc., decreased 55 metabolites such as flavin adenine dinucleotide and xanthine, and metabolic pathway enrichment analysis found that CS was mainly involved in purine metabolism) and significantly up-regulating the expression level of the intestinal uric acid excretion transporter ATP-binding cassette subfamily G member 2 (ABCG 2) (Zhu et al., 2023); 3. CS demonstrated the ability to impede the progression of epithelial–mesenchymal transition (EMT) in renal tubular cells of rats with hyperuricemia nephropathy and HUA-HK 2 cells by inhibiting the NF-κB/Snail signaling pathway, thereby alleviating renal injury and showcasing significant anti-hyperuricemia and nephroprotective properties of CS (Wu et al., 2023).

Based on the preceding explanation, it is evident that the hyperoxic diterpenoids and phenolic acids in CS as effective components have shown promising potential for the treatment of hyperuricemia and gout via multi-target and multi-pharmacodynamic mechanisms.

### 4.6 Nephroprotective

The EE extract of CS demonstrated potent nephroprotective effect via lowering melamine in mice renal, impeding stone formation, and mitigating renal injury (Zhang J. et al., 2021). A variety of flavonoids in CS that exhibited robust renal protection effects, with trials demonstrating substantial reductions in proteinuria, serum creatinine, and urea nitrogen in rats with acute renal failure model, as well as decreased oxidative stress. The therapeutic effect of CS on acute renal failure may be related to the nephroprotective effect mediated by activation of the ERK/CT-1 pathway (Guo et al., 2019a). Additionally, these flavonoids stimulate renal tubular epithelial cell proliferation in acute renal failure, curtail apoptosis, modify oxidative stress levels, suppress pro-apoptotic protein expression, and enhance renal tubular epithelial cell repair in acute renal failure (Guo et al., 2019b).

### 4.7 Other effects

Two novel phenolic acid Helisterculins C and D (**190** and **191**), derived from CS *in vitro*, demonstrated neuroprotective potential against 6-OHDA-mediated SH-SY5Y cell death, with IC50 values of 17.4 and 21.3 μM, respectively (Zhou et al., 2017). In addition, research indicated that 2-caffeoyl-L-tartaric acid and rosmarinic acid outperformed acarbose in reducing *α*-glucosidase activity *in vitro* (Zhang, 2017). In clinical practice, CS potent therapeutics towards urinary tract stones, with EAE extract preventing kidney stones via mitigating oxidative stress and inflammation amplified by glycerophospholipid metabolism (Zhu et al., 2021).

The research mentioned above highlights how pharmacological studies on CS not only validate its traditional uses but also uncover its potential applications in treating other diseases. The key components in CS, such as diterpenoids, flavonoids, and phenolic acids, play a crucial role in its pharmacological effects. These compounds enable CS to exhibit multiple therapeutic targets and mechanisms in combating inflammation, oxidative stress, tumors, hyperuricemia, gout, and nephroprotective. However, while CS has shown promising anti-tumor effects *in vitro*, further research is needed to explore its efficacy *in vivo* and understand the underlying pharmacological mechanisms.

## 5 Quality control

The current and previous editions of the Chinese Pharmacopoeia exclude CS from their permissible content, and the quality control measures for CS have traditionally relied on outdated provincial or local criteria, leading to inconsistencies in quality control research due to the absence of standardized guidelines. In order to obtain stable and reliable quality control methods to promote the development and utilization of CS, researchers were committed to enhancing and refining essential technologies in quality control research, including qualitative identification, quantitative analysis, and fingerprint analysis.

### 5.1 Qualitative identification

Qualitative identification is a significant channel to ensure the correctness, excellent quality, safety and effectiveness of medicinal materials, and Morphological identification, microscopic identification and thin layer chromatography (TLC) identification are momentous means for qualitative identification of medicinal materials (Wang et al., 2022). Jiao et al. used traditional methods to identify the traits of CS, and observed the cross-section of leaves and stems and the microscopic characteristics of powder of CS by paraffin sectioning method, which provided more detailed and comprehensive characteristics for the identification of CS (Jiao and Feng, 2013). In the study, researchers employed danshensu, rosmarinic acid, and ursolic acid as marker ingredients for TLC identification, replacing the conventional reference materials typically used in this analytical technique. Through systematic investigation of single factors and adaptability of the system, a straightforward, practical, reproducible, and specific TLC identification method was developed for quality control of CS (Gu, 2023).

### 5.2 Quantitative analysis

Ursolic acid is one of the main chemical substances that play pharmacological roles in CS, and some preparations have recognized it as a reference material for quality testing and control. Huang and colleagues (Huang et al., 2019) determined the ursolic acid content in the alcohol extract of CS using HPLC under the following optimal conditions: mobile phase: acetonitrile-5% tetrahydrofuran with 0.1% phosphoric acid in a ratio of 68:32; wavelength: 203 nm; column temperature: 33°C; flow rate: 1 mL/min; injection volume: 20 *μ*L. The average recovery was 101.11% (RSD = 2.04%). This technique demonstrated high resolution and reduced analysis time, thereby offering a scientific foundation for the quality assessment of ursolic acid in CS alcohol extracts. In a separate investigation, the levels of danshensu and rosmarinic acid in CS were analyzed using HPLC. The analysis employed a Venusil C_18_ column with a mobile phase gradient elution consisting of acetonitrile and a 0.1% phosphoric acid aqueous solution, flowing at a rate of 1 mL/min. Detection was performed at a wavelength of 210 nm, with the column maintained at 30°C and an injection volume of 5.0 *μ*L. The quantification of danshensu and rosmarinic acid ranged from 1.75 to 56 *μ*g and 15–480 *μ*g, respectively, demonstrating a strong linear correlation. This straightforward, consistent, and dependable method offered valuable insights for the quality control assessment of CS medicinal materials (Gu, 2023).

Chinese materials display varying qualities depending on their origin, harvesting season, climate, and other variables, influencing drug efficacy, with establishing an appropriate quality standard is vital for Chinese herbal quality standards. A noteworthy finding revealed that the extracts of CS from the same region, which summer harvests contained higher levels of total polyphenols, total flavonoids, and antioxidant capacity, compared to autumn, while rosmarinic acid content varied depending upon region and season (Xue et al., 2016). Moreover, in order to evaluate the difference in quality between CS with purple corollas and white corollas, the researchers employed a colorimetric approach to ascertain the levels of total phenolic acids and total flavonoids. Furthermore, the quantities of caffeic acid, rosmarinic acid, and eupatorin were determined using HPLC. The results revealed that the levels of total phenolic acids, total flavonoids, and eupatorin were higher in CS with purple corollas compared to those CS with white corollas, while the quantities of caffeic acid and rosmarinic acid were found to be similar (Li et al., 2023).

Dao-di medicinal materials are high-quality Chinese herbal products, considered the first choice in Traditional Chinese Medicine (TCM) due to their historical significance and superior efficacy. CS primarily grows in Yunnan, Guangxi, Hainan, other southern regions of China, especially the CS which from Yunnan has always had a well reputation in traditional applications and modern clinical research. Through origin analysis, it has been determined that CS from Yunnan exhibits superior quality, most notably the CS which was from Xishuangbanna with highest rosmarinic acid content compared with other origins (Lan et al., 2017).

### 5.3 Fingerprint analysis

The fingerprint analysis technique in traditional Chinese medicine enables a comprehensive assessment of the chemical composition present in traditional Chinese medicine and its formulations, facilitating a detailed description and evaluation of the overall drug quality. In a research study, through statistical analysis of the fingerprints of 13 batches of CS medicinal materials, 12 common peaks were identified, and two common components (danshensu and rosmarinic acid) were identified by comparison with control products. The results of similarity analysis indicated that the similarity among the 13 batches of CS medicinal materials ranged from 0.698 to 0.997. Notably, the similarity among the fingerprints of 12 batches exceeded 0.970, with the exception of the S4 sample, which exhibited a similarity lower than 0.970 when compared to the control fingerprint (Gu, 2023). In recent times, there has been notable progress in ultra-performance liquid chromatography (UPLC) technology, enhancing the efficiency of liquid chromatography separations by elevating resolution, sensitivity, and analysis speed beyond what is achievable with conventional HPLC techniques. In accordance with this, Guo et al. (2019) conducted an examination of the resemblance among the predominant peaks present in 8 distinct batches of CS medicinal materials using the UPLC technique in conjunction with the “Chromatographic Fingerprint Similarity Evaluation System of Traditional Chinese Medicine 2004A Edition.” They established the fingerprints for these 8 batches of CS medicinal materials, identified 15 distinctive common peaks, and ascertained the constituents of 5 chromatographic peaks (caffeic acid, rosmarinic acid, salvianolic acid B, sinsensetin and eupatorine) through comparison with a reference substance. For the fingerprint analysis of volatile components in CS, Liu et al. (2016) utilized GC-MS in conjunction with programmed temperature retention index technology to examine 12 batches of CS samples. They established a volatile components fingerprint using GC-MS and identified 13 common chromatographic peak components, predominantly comprising alcohols, ketones, naphthalene, alkanes, acids, aldehydes, esters, and other compounds. Furthermore, the “Chromatographic Fingerprint Similarity Evaluation System Software for Traditional Chinese Medicine” (2004A Edition) was employed to conduct a more in-depth analysis of the fingerprint, revealing a similarity coefficient exceeding 0.999 among the 12 batches of CS samples. This approach offers a comprehensive depiction of the volatile components present in CS, characterized by its specificity, thus serving as a valuable tool for ensuring the quality control of volatile components in this medicinal material.

CS has demonstrated positive outcomes in the treatment of urinary system inflammation, gout, and various other medical conditions. Numerous clinical cases have supported its efficacy in treatment. Therefore, the implementation of standardized quality control measures is imperative in ensuring its safe and effective application. The current body of research provides some valuable insights for the quality control of CS to a limited extent. However, the overall quality control standards for CS are typically inadequate, primarily focusing on qualitative identification and content determination of a limited number of components. These compounds are not exclusive to CS, leading to a lack of credibility in contemporary quality control studies. Future research should prioritize qualitative identification and content determination of unique constituents within CS, while also considering the establishment of a quality evaluation and control system that integrates fingerprinting and multi-component content determination.

## 6 Clinical applications

CS was prevalent in managing urinary tract stones, infections, nephrotic syndrome, renal failure, hyperuricemia, etc. Individually or in conjunction, it exhibited commendable efficacy, with minimal suspicious adverse reactions. The detailed data is delineated in Table 8.

**TABLE 8:** Clinical application research studies of *Clerodendranthus spicatus*.

Modes of medication	Types of disease	Authors (Years)	Study design control type	Dosage of study drug and control drug	Number of subjects; age	Duration of treatment	Diagnosis inclusion criteria	Exclusion criteria	Therapeutic effect
Single-drug administration	Chronic glomerulonephritis albuminuria	Xie et al. (2012)	Positive control	**1** CS **2** Benazepril	43 in treatment group (23 men and 20 women, 31–58 years) 42 in Control group (24 men and 18 women, 27–62 years)	3 months	Chronic glomerulonephritis albuminuria	1 Failure to take medication according to regulations or incomplete data affects the observation of efficacy. 2 Patients with severe malnutrition, renal insufficiency, and serious damage to the heart, liver, and hematopoietic system.	The total effective rate was 90.70% in the treatment group and 88.10% in the control group. The clinical efficacy of the treatment group was comparable to that of the control group
	Chronic glomerulonephritis	Xie et al. (2013)	Positive control	**1** CS **2** Benazepril	63 in treatment group (33 men and 30 women, 32–61 years) 52 in Control group (34 men and 28 women, 27–64 years)	12 weeks	Albuminuria, Albuminuria is combined with hematuria	1.Patients with purpuric nephritis and secondary kidney disease of systemic wolf erythematosus. 2.Patients with liver cirrhosis, tumors, diabetes and other systemic diseases.	The overall response rate was 84.1% in the treatment group and 82.3% in the control group. There was no statistically significant difference in both groups
	Chronic nephritis	Xie (2018)	Blank control	**1** Basic treatment + CS **2** Basic treatment	44 in treatment group (28 men and 16 women, mean age 49.1 years) 44 in Control group (30 men and 14 women, mean age 47.6 years)	4 weeks	Compensated early chronic nephritis	1 Renal failure, serum creatinine≥133 *μ*mol/L. 2 Secondary nephritis. 3 Lactating and pregnant women. 4 Mental and cognitive disorders. 5 Subjects who were allergic CS	The control rate and apparent efficiency of the observation group were higher than those of the control group, and the overall efficacy of the observation group was better than that of the control group
	Chronic renal insufficiency in polycystic kidney disease	Huang (2000)	Blank control	**1** CS **2** Symptomatic management	20 patients (8 men and 12 women) of 45–76 years	6 years	Chronic renal insufficiency in polycystic kidney disease	Not reported	CS delayed the deterioration of kidney function in patients with polycystic kidney disease and had no toxic side effects
	Urinary tract infections	Liu et al. (2000)	Not reported	CS	38 patients (6 men and 32 women) of 17–72 years	3 weeks	Urine examination revealed red blood cells and white blood cells. Mid-urine culture revealed growth of pathogenic bacteria.	Not reported	The total effective rate of CS treatment was 90%, and CS had shown unique efficacy in the treatment of urinary tract infections
	Urinary tract stones	Huang et al. (1999)	Randomized, double-blind observation	**1** CS **2** Placebo	126 patients (66 men and 60 women) of 6–60 years and mean age 47.6 years	2 weeks	Urinary tract stones, haematuria	Not reported	The recovery rate and total effective rate of the CS group were significantly higher than those of the control group
Combination medications	Chronic renal failure	Xu and Song (2007)	Randomized blank control	**1** treatment group: conventional Western medicine treatment + Chongcao Shencha Capsule (Contains CS) **2** Control group: conventional Western medicine treatment	A total of 60 subjects were randomly divided into treatment group and control group. There were 30 cases in each group, and 10 cases in each group included CKD stages 3–5	3 months	Chronic glomerulonephritis, Diabetic nephropathy, Hypertensive nephropathy, Chronic pyelonephritis	1 Patients with CKD stage 1 – 2. 2 Complications such as severe anemia, infection, heart failure, encephalopathy and hematopoietic system disease 3 Those with poor compliance	The clinical symptoms were improved, and the symptoms of the treatment group were significantly improved, especially in CKD stage 3 and CKD 4, which were better than that of the control group
Combination medications	Chronic renal Failure	Yu et al. (2007)	Randomized blank control	**1** treatment group: conventional Western medicine treatment + Cordyceps and CS formula (Contains CS) **2** Control group: conventional Western medicine treatment	42 patients (18 men and 26 women) of 29–65 years and mean age 48.67 years.	3 months	Chronic glomerulonephritis, Diabetic nephropathy, Hypertensive nephropathy, Interstitial nephritis, Chronic pyelonephritis	1 Patients with CKD stage 1-2. 2 Patients with CKD stage 5. 3 Patients with severe anemia, infection, heart failure, encephalopathy and other complications that are difficult to control. 4 Those who cannot cooperate with the treatment and have poor compliance	The total effective rate of the treatment group was 76.19%, which was better than that of the control group (52.38%)
	Chronic renal failure	Dai (2007)	Randomized positive control	**1** Chongcao shencha fang (Contains CS) **2** Uretoxin clear granules	22 in treatment group (Gender unknown, mean age 51.14 years) 22 in Control group (Gender unknown, mean age 53.86 years)	3 months	Chronic glomerulonephritis, Chronic pyelonephritis, Diabetic nephropathy,Hypertension renal arteriole sclerosis, Hyperuric acid nephropathy	Patients with acute and chronic infections and severe hyperparathyroidism, as well as serious primary diseases such as heart, brain, liver and hematopoietic system, systemic lupus erythematosus, nephritis, obstructive nephropathy, etc. Those who were allergic or allergic to multiple drugs.	There was a significant difference in disease efficacy between the two groups (*P*<0.05) by Ridit analysis, and the treatment group was better than the control group
	Diabetic nephropathy	Song et al. (2009a)	Randomized positive control	**1** Cordyceps and CS formula (Contains CS) **2** Sodium Fosinopril Tablets	58 patients (27 men and 31 women) of 42–73 years and mean age 52.6 years.	8 weeks	Diabetic nephropathy	Not reported	The overall response rate was 76.7% in the treatment group compared to 46.3% in the control group. The treatment group was significantly better than the control group in reducing urine protein and improving renal function (*P*<0.05)
	Diabetic nephropathy	Song et al. (2009b)	Randomized positive control	**1** Cordyceps and CS capsules (Contains CS) **2** Sodium Fosinopril Tablets	30 in treatment group (17 men and 13 women, mean age 45.77 years) 30 in Control group (16 men and 14 women, mean age 46.83 years)	16 weeks	Diabetic nephropathy	1 Pregnant and lactating women. 2 Those who are allergic or allergic to a variety of drugs. 3 Patients with serious primary diseases and mental illnesses such as heart, brain, liver, and hematopoietic system.	There were no adverse reactions in the treatment group and the control group. The total response rate was 83.3% in the treatment group and 67.8% in the control group. The urine albumin excretion rate, 24-hour urine protein quantification, serum creatinine and urea nitrogen changes in the treatment group were better than those in the control group
	Chronic prostatitis	Wang et al. (2014)	Not reported	CS recipe (Contains CS)	42 patients (Gender unknown) of 20–50 years and mean age 32 years.	6 weeks	Chronic prostatitis	1 Those who were allergic to this drug. 2 Patients with acute prostatitis, prostate tuberculosis, prostate cancer, neurogenic bladder, bladder tumor, urinary tract stones, varicocele and epididymitis. 3 Patients with severe diabetes, cardiovascular disease, liver and kidney function abnormalities. 4 Patients with poor compliance.	The total effective rate of CS recipe was 93.33%. The patient had no obvious discomfort during the medication
Combination medications	Chronic glomerulonephritis	Liu (2018)	They were randomly divided into treatment group and control group	**1** Turbidity Tang (Contains CS) **2** Nephritis rehabilitation tablets	30 in treatment group (Gender and age unknown) 30 in Control group (Gender and age unknown)	8 weeks	Chronic glomerulonephritis	1 Patient<age 18 years, or >70years old. 2 Pregnant or lactating women. 3 Those with abnormal renal function tests 4 Those who have recently used hormonal drugs within 2 months of treatment; 5 Patients with severe hypertension, heart failure, infection, water and electrolyte disorders, acid-base balance disorders, and serious primary diseases such as heart, brain, liver and hematopoietic system.	The total effective rate of the treatment group was 93.33%, and that of the control group was 80.00%, and the total clinical efficacy of the experimental group was better than that of the control group.
	Urinary stones	Wang (1998)	Positive control	**1** Shencha Xiaoshi decoction (Contains CS) **2** Stone removal powder	60 patients (42 men and 18 women) of 23–52 years.	2 years	Kidney stones, Ureteral stones, Bladder stones	Not reported	The effective rate of the treatment group was 93.33%, and the effective rate of the control group was 16.66%, and the stimulation of the gastrointestinal tract of CS stone decoction was significantly less than that of the control group
	Urinary stones	Fang (2008)	Not reported	Shencha paishi decoction (Contains CS)	50 patients (32 men and 18 women) of 23–60 years.	20 days	Kidney stones, Ureteral stones, Bladder stones	Not reported	30 cases were cured, 15 cases were improved, and 5 cases were not cured, with a total effective rate of 90%。
	Hyperuricemia	Zhang (2020)	The numeric random table method was divided into the experimental group and the control group	**1 S**henchayin (Contains CS) **2** Control diet	61 patients (42 men and 19 women) of 22–63 years and mean age 36.3 years.	3 months	Hyperuricemia	1 Secondary HUA caused by glomerular disease, lead poisoning, hematological disease, tumor radiotherapy, chemotherapy, etc 2 Patients with severe cardiovascular and cerebrovascular diseases, acute and uncontrollable diseases, chronic diffuse connective tissue diseases, xanthine urethral deposition, untreated thyroid diseases, severe hypertension or diabetes mellitus that have not been effectively controlled after treatment, hematopoietic system, digestive system, and severe liver and kidney insufficiency	The total effective rate of the treatment group (81.25%) was higher than that of the control group (20.69%), and the liver function indexes, renal function indexes and blood lipid indexes of the treatment group were better than those of the control group.
Combination medications	Hyperuricemia	He and Xu (2018)	Positive control	**1** Compound CS mixture (Contains CS) **2** Allopurinol	30 in treatment group (Gender and age unknown) 30 in Control group (Gender and age unknown)	4 weeks	Hyperuricemia	Not reported	The total effective rate of the treatment group was 83.33%, and that of the control group was 86.67%, and the Compound CS mixture had a significant effect on reducing blood uric acid in the treatment of asymptomatic hyperuricemia
	Hematuria	Yu (2012)	Not reported	XueNiaoAnPian (Contains CS)	148 patients (50 men, mean age 46 years and 98 women, mean age 38 years)	2 weeks	Hematuria from all causes	Not reported	Among the 148 subjects, 115 cases were effective and 33 cases were effective
	Nephrotic syndrome	Huang (1999)	They were randomly divided into treatment group and control group	**1** prednisone + CS **2** prednisone	33 in treatment group (19 men and 14 women, mean age 26.1 years) 30 in Control group (18 men and 12 women, mean age 25.3 years)	3 months	Nephrotic syndrome	Not reported	The total effective rate of the treatment group was better than that of the control group (*P*<0.05), and the recurrence rate of the treatment group was significantly better than that of the control group (*P*<0.01).

## 7 Conclusions and future perspectives

This review summarized the most recent research on CS, focusing on its chemical constituents, pharmacological activity, quality control and clinical applications. The insights presented in this review serve as a valuable resource for guiding future investigations and advancements in this field. CS is a widely recognized herbal remedy in China and various Southeast Asian nations. It is extensively documented in ancient Chinese materia medica books, boasting a rich history of use and renowned efficacy in treating urological ailments. This reputation has piqued the curiosity of researchers worldwide, prompting comprehensive investigations into its properties and applications. Presently, over 300 compounds have been isolated from CS, expanding its pharmaceutical applications from the urinary system to various physiological systems and treatments for ailments such as the circulatory and nervous systems, with exhibiting notable pharmacological activities in anti-inflammatory, antibacterial, antioxidant, antitumor, anti-gout, renal protection, neuroprotection, and hypoglycemia, notably the active components such as total flavonoids, phenolic acids, and diterpenoids with high oxygen content, are hypothesized to be pivotal in anti-inflammatory, renal protection, neuroprotection, and urate-lowering activities. Preparations of traditional Chinese medicine that include CS, as well as CS on its own, have demonstrated positive outcomes in clinical interventions for conditions such as urinary tract infection, urinary tract stones, nephritis, renal insufficiency, chronic renal failure, diabetic nephropathy, hyperuricemia, and other related diseases. The favorable pharmacological activities and clinical efficacy can be attributed to the diverse therapeutic effects of compounds or constituents found in CS, which act on multiple targets and pathways.

Despite the considerable body of scientific research conducted on CS, there remain certain constraints that require immediate disposition. First of all, research on the phytochemical and pharmacological properties of CS has primarily concentrated on phenylpropanoids, flavonoids, and terpenoids, with limited emphasis on other compounds such as polysaccharides and saponins. Furthermore, existing pharmacological investigations have predominantly provided superficial assessments of drug efficacy, lacking in-depth exploration of the mechanisms underlying the anti-inflammatory, antibacterial, neuroprotective, and other pharmacological effects associated with CS. Hence, it is imperative to conduct further research on the pharmacodynamic material basis and mechanisms of action of CS in the treatment of significant medical conditions. This will establish a robust groundwork for CS as a therapeutic agent for a broader range of diseases. Secondly, despite the existence of numerous quality control studies focusing on CS, these studies often fail to select the signature components of CS as the control objects, thus rendering the current research on quality standards inconclusive. Given the intricate and diverse nature of the components found in CS, as well as their pharmacological activity being influenced by the synergistic effects of multiple components, a mere examination of individual components is insufficient for evaluating the quality of an active ingredient. Therefore, it is feasible to establish a quality evaluation and control system that integrates fingerprinting and multi-component content determination. Enhancing the quality control system for CS necessitates not only the thorough investigation by scholars in the field, but also the policy backing from relevant governmental bodies. This entails the development of unified and scientifically sound quality control standards through comprehensive and systematic research, as well as the timely inclusion of CS in the Chinese Pharmacopoeia. Such efforts are crucial for the advancement and effective utilization of CS. Moreover, clinicians predominantly rely on ancient Chinese materia medica books and personal experience when utilizing CS in patient treatment. While CS has demonstrated effectiveness, there remains a deficiency in comprehensive preclinical assessments of its safety and efficacy. Addressing this gap necessitates collaborative efforts across various disciplines and departments to establish a more robust scientific foundation supporting the secure, efficient, and consistent application of CS in clinical practice.

In anticipation of the future, it is recommended that pertinent governmental agencies enhance the promotion and policy supporting of CS to broaden its impact. Scholars are advised to draw inspiration from traditional Chinese medicine and ethnic medicinal practices, while integrating cutting-edge scientific technologies and research methodologies to explore more compounds with significant activity in CS and improve their pharmacodynamic mechanism research. Furthermore, it is imperative to conduct spectrum-effect relationship studies on CS, develop robust quality control criteria focusing on the active pharmaceutical ingredients and characteristic components present in CS. By integrating the quality of CS with its therapeutic efficacy to offer a more comprehensive theoretical and practical foundation for utilizing CS in disease prevention and treatment.

## Glossary

ABCG 2: ATP-Binding Cassette subfamily G member 2
ADA: Adenosine Deaminase
BCB: *β*-carotene Bleaching
CE: Chloroform Extract
CS: *Clerodendranthus spicatus*
CBM: Chinese Biomedical Database
CN: Calcineurin
CNKI: China National Knowledge Infrastructure
CKD: Chronic Kidney Disease
DPPH: 2,2-diphenyl-1-picrylhydrazyl
EAF: Ethyl Acetate Fraction
EAE: Ethyl Acetate Extract
EE: Ethanol Extract
EMT: Epithelial–Mesenchymal Transition
GC-MS: Gas Chromatography–Mass Spectrometry
GLUT 9: Glucose Transporter 9
HUA: Hyperuricemia
HPLC: High Performance Liquid Chromatography
_**L**_ **-NMMA**: N^G^-Monomethyl-_L_-Arginine
MDA: Malondialdehyde
NFAT: Nuclear Factor of Activated T-cells
NO: Nitric Oxide
OAT 1: Organic Anion Transporter 1
OAT 3: Organic Anion Transporter 3
OSE: *Orthosiphon stamineus* Ethyl acetate extract
PE: Petroleum Ether extract
PO: Potassium Oxazinate
TCM: Traditional Chinese Medicine
TLC: Thin Layer Chromatography
UPLC: Ultraperformance Liquid Chromatography
UA: Ursolic Acid
URAT 1: Urate Transporter 1
WE: Water Extract
XOD: Xanthine Oxidase
